# Microencapsulation of Lactic Acid Bacteria Improves the Gastrointestinal Delivery and *in situ* Expression of Recombinant Fluorescent Protein

**DOI:** 10.3389/fmicb.2018.02398

**Published:** 2018-10-05

**Authors:** Nina D. Coelho-Rocha, Camila P. de Castro, Luis C. L. de Jesus, Sophie Y. Leclercq, Savio H. de Cicco Sandes, Alvaro C. Nunes, Vasco Azevedo, Mariana M. Drumond, Pamela Mancha-Agresti

**Affiliations:** ^1^Laboratório de Genética Celular e Molecular, Instituto de Ciências Biológicas, Departamento de Biologia Geral, Universidade Federal de Minas Gerais, Belo Horizonte, Brazil; ^2^Kroton Educacional, Faculdade Pitágoras, Contagem, Brazil; ^3^Laboratório de Inovação Biotecnológica, Fundação Ezequiel Dias, Belo Horizonte, Brazil; ^4^Laboratório de Genética Molecular de Protozoários Parasitas, Instituto de Ciências Biológicas, Departamento de Biologia Geral, Universidade Federal de Minas Gerais, Belo Horizonte, Brazil; ^5^Centro Federal de Educação Tecnológica de Minas Gerais, Coordenação de Ciências, Belo Horizonte, Brazil

**Keywords:** recombinant *Lactococcus lactis*, sodium alginate encapsulation, mCherry reporter protein, pExu vector, DNA delivery

## Abstract

The microencapsulation process of bacteria has been used for many years, mainly in the food industry and, among the different matrixes used, sodium alginate stands out. This matrix forms a protective wall around the encapsulated bacterial culture, increasing its viability and protecting against environmental adversities, such as low pH, for example. The aim of the present study was to evaluate both *in vitro* and *in vivo*, the capacity of the encapsulation process to maintain viable lactic acid bacteria (LAB) strains for a longer period of time and to verify if they are able to reach further regions of mouse intestine. For this purpose, a recombinant strain of LAB (*L. lactis* ssp. *cremoris* MG1363) carrying the pExu vector encoding the fluorescence protein mCherry [*L. lactis* MG1363 (pExu:*mCherry*)] was constructed. The pExu was designed by our group and acts as a vector for DNA vaccines, enabling the host cell to produce the protein of interest. The functionality of the pExu:*mCherry* vector, was demonstrated *in vitro* by fluorescence microscopy and flow cytometry after transfection of eukaryotic cells. After this confirmation, the recombinant strain was submitted to encapsulation protocol with sodium alginate (1%). Non-encapsulated, as well as encapsulated strains were orally administered to C57BL/6 mice and the expression of mCherry protein was evaluated at different times (0–168 h) in different bowel portions. Confocal microscopy showed that the expression of mCherry was higher in animals who received the encapsulated strain in all portions of intestine analyzed. These results were confirmed by qRT-PCR assay. Therefore, this is the first study comparing encapsulated and non-encapsulated *L. lactis* bacteria for mucosal DNA delivery applications. Our results showed that the microencapsulation process is an effective method to improve DNA delivery, ensuring a greater number of viable bacteria are able to reach different sections of the bowel.

## Introduction

The intestinal microbiota is composed of a huge diversity of ecological niches, encompassing more than 50 genera of bacteria (Holdeman et al., 1976) with different characteristics: harmful, beneficial, or both. Among the bacteria with beneficial activity, the LAB can be highlighted. These are Gram-positive, saccharolytic, rod shaped, non-sporulating bacteria with low-GC content genomes, that could reside in the large intestine (Salminen et al., 1998). The majority of LAB have a ‘Generally Recognized as Safe’ (GRAS) status according to the United Sates Food and Drug Administration (US FDA) and fulfill of the Qualified Presumption of Safety (QPS) notion criteria developed by the European Food Safety Authority (EFSA).

Some strains are classified as probiotic, promoting them to be beneficial for consumer health (Sanders et al., 2013). This intrinsic advantage of some LAB, as well as the easiness to design them in recombinant lactic acid bacteria (rLAB) makes them a good alternative to systemic delivery systems compared with other mucosal delivery vehicles such as liposomes and attenuated pathogens. For these reasons, over the past two decades, LAB has been intensively studied as potential carriers of compounds with therapeutic or prophylactic effects at mucosal surface (Wells and Mercenier, 2008; Bermúdez-Humarán, 2009; Zurita-Turk et al., 2014; Souza et al., 2016; Mancha-Agresti et al., 2017). In this context, plasmid-cured strains of *L. lactis* ssp. *lactis* IL1403 and *L. lactis* ssp. *cremoris* MG1363, whose genomes were sequenced in 1999/2001 and 2007, respectively, are the strains most commonly used (Bolotin et al., 2001; Wegmann et al., 2007; Linares et al., 2010) for production of recombinant molecules in mucosal vaccination (Wells and Mercenier, 2008). In addition, interest in genus *Lactobacillus* has also increased (Li et al., 2007; Hongying et al., 2014; Allain et al., 2016). In DNA delivery system the eukaryotic host cells express antigens encoded by the vaccine. Epitopes exposed by the recombinant protein (antigen) are very similar to those in their native form (Běláková et al., 2007), consequently they are presented to the immune system in an analogous form found in nature.

Oral administration of rLAB producing/coding therapeutics proteins have the benefit of direct proteins delivery *in situ*, avoiding degradation either by enzymatic action or adverse conditions encountered in the stomach such as severe acid challenges. Also, rLAB could be protect against stress elicited by the exposure to high bile and salt conditions of the intestine faced during transit through the gastrointestinal tract (Drouault et al., 1999). On the other hand, studies have shown that the percentage of viable bacteria that reaches the gut may be considerably lower compared to the number of administered bacteria (Charteris et al., 1998; Cook et al., 2012), due to above mentioned stress conditions.

To circumvent this drawback, bacterial microencapsulation technique using polymer matrix offers adequate conditions to reduce bacterial loss, in the gastrointestinal tract, as well as in food products (Fávaro-Trindade and Grosso, 2002). The most widely used matrix for microencapsulation is sodium alginate, a non-toxic and biocompatible polymer which is able to establish a versatile matrix which protects active components, as well as probiotic microorganisms sensible to pH, heat, oxygen, and other factors (Goh et al., 2012). Some studies report an increase of up to 80–95% survival of encapsulated probiotics with alginate beads (Sheu and Marshall, 1993; Jankowski et al., 1997; Krasaekoopt et al., 2003). Lower bacteria population reduction during exposure to simulated gastric environment and bile solution (Picot and Lacroix, 2004) are some features improved by encapsulation of probiotics in alginate. Encapsulation in alginate of *Bifidobacterium longum* as well as, *Lactobacillus rhamnosus* improved survival at acid pH (pH 2.0) up to 48 h and also, death rate decreased proportionately with increased alginate concentrations (2–4%) and bead size (Lee and Heo, 2000; Goderska et al., 2003).

In previous work, our group developed a new vector (pExu – Extra Chromosomal Unit) to be used as a DNA delivered by *Lactococcus lactis* and *Lactobacillus* strains. We showed eGFP expression (Enhanced Green Fluorescent Protein), by enterocytes in the duodenal region between 12 up to 72 h after oral administration of the recombinant strain *L. lactis* (pExu:*egfp*) (Mancha-Agresti et al., 2016). Nevertheless, tissue auto fluorescence is in the same GFP protein wavelength, hindering tissue analysis and eGFP expression detection in the lower bowel region. Nowadays, there are many fluorescent proteins available (Shaner et al., 2005; Ai et al., 2008; Day and Davidson, 2009). Among the best red members in the fruit series, the RFP (Red Fluorescent Protein) mCherry, can be highlighted. This protein is obtained from successive modifications of the *Discosoma* ssp. fluorescent protein DsRed, and it has longest excitation and emission wavelengths (587/610 nm, respectively) with shortest maturation time (Patterson et al., 2001; Shaner et al., 2004; Jach et al., 2006) in comparison with other RFPs. Proteins that emit fluorescence in red zone are more desirable for cellular studies than those which emit in green or blue zones because red light presents less dispersion allowing deep tissue penetration (Nienhaus and Wiedenmann, 2009), therefore increasing sensibility (Deliolanis et al., 2008).

The aim of this study was to encapsulate the *L. lactis* ssp. *cremoris* MG1363 strain carrying the pExu vector encoding RFP *mCherry* gene in alginate matrix and evaluate potential delivery across gut portions after oral administration by comparing non-encapsulated and encapsulated bacteria.

## Materials and Methods

### Bacterial Strains and Plasmids

Bacterial strains and plasmids used in this study are listed in **Table 1**. *E. coli* Top10 strain was grown in Luria-Bertani (LB) medium (Acumedia, Lansing, MI, United States) at 37°C with vigorous shaking. *L. lactis* ssp. *cremoris* MG1363 was grown statically at 30°C in M17 medium (Sigma-Aldrich, St. Louis, MO, United States) supplemented with 0.5% glucose (w/v) (Labsynth, São Paulo, Brazil) (G-M17). The Erythromycin antibiotic (Sigma-Aldrich) was added at the indicated concentration as necessary; 500 μg/mL for *E. coli* and 125 μg/mL for *L. lactis*. Pure cultures of bacteria were kept as stock cultures in 40% glycerol (v/v) (Sigma-Aldrich) for *E. coli* and 25% glycerol (v/v) for *L. lactis* at −80°C.

**Table 1:** Bacterial strains and plasmids used in this work.

Bacterial strain	Characteristics	Source
*Escherichia coli* TOP10	*E. coli* K-12-derived strain; F-mcrA 1 (mrr-hsdRMS-mcrBC) 8 80lacZ1M15 1lacX74 nupG recA1 araD139 1 (ara-leu)7697 galE15 galK16 rpsL (StrR) endA1 λ	Invitrogen
*Escherichia coli* TOP10 (*pTP:mCherry*)	*E. coli* TOP10 carrying the pTP:*mCherry* plasmid; Km^r^	This work
*Escherichia coli* TOP10 (*pExu:mCherry*)	*E. coli* TOP 10 carrying the pExu:*mCherry* plasmid; Ery^r^	This work
*Lactococcus lactis* MG1363	*L. lactis* ssp. *cremoris*	Gasson, 1983
*Lactococcus lactis* MG1363 (pExu:*mCherry*)	*L. lactis* MG1363 carrying the pExu:*mCherry* plasmid; Ery^r^	This work

***Plasmids***	***Characteristics***	**Source**

Zero Blunt^®^ TOPO^®^	Cloning vector (KmR, ccdB gene fused to the C-terminus of the LacZα fragment)	Invitrogen
pXJM19:*mCherry*	ori colE1, oricg, ptac, *mCherry*, Cm^r^	Ott et al., 2012
pTP:*mCherry*	TOPO^®^ vector with the *mCherry* ORF; Km^r^	This work
pExu:*mCherry*	pCMV/Ery^r^/RepA/RepC/mCherry	This work

*Plac: promoter of operon lac; Km^r^: gene that confers resistance to kanamycin; pUc ori: origin of replication; ccdB: lethal gene; ZeoR: gene resistance to zeomycin, ori ColE1: origin of replication for E. coli; Amp: gene that confers ampicillin resistance; pCMV: cytomegalovirus promoter; Ery: gene that confers erythromycin resistance; RepD and RepE: origins of prokaryotic replication for L. lactis and Lactobacillus; egfp: coding sequence of enhancer fluorescent green protein; Cm: gene that confers resistance to chloramphenicol; mCherry: coding sequence of red fluorescent monomeric protein.*

### DNA Manipulations

The protocols of DNA manipulation were performed following Sambrook protocols (Green and Sambrook, 2012) with some modifications. For plasmid DNA extraction from *L. lactis*, to prepare the protoplasts, TES buffer (25% sucrose, 1 mM EDTA, and 50 mM Tris-HCl pH 8) containing lysozyme (Sigma- Aldrich) (10 mg/mL) was added for 1 h at 37°C. Restriction enzymes were used as recommended by suppliers. Transformation of *L. lactis* was performed by electroporation (2,400 V, 25 μF capacitance and 200 Ω of resistance). *L. lactis* transformants were plated on G-M17 agar plates containing erythromycin and were counted after 48 h incubation at 30°C, whereas *E. coli* transformants were plated onto LB agar plates containing the required antibiotic for 24 h at 37°C.

### pExu:*mCherry* and *L. lactis* ssp. *cremoris* MG1363 (pExu:*mCherry*)

The ORF of reporter gene *mCherry* was amplified by PCR technique using Hot Start^®^ high-fidelity DNA Polymerase (Qiagen, Hilden, Germany). Oligonucleotides primers used for mCherry were: 5′ ggcGCGGCCGCAATGGTGAGCAAGGGCGAGG 3′ (forward) and 5′ ggcCTCGAGTTACTTGTACAGCTCGTCCATGC 3′ (reverse). Artificial restriction sites of *Not*I and *Xho*I enzyme and also, customized Kozak sequence were added in the forward oligonucleotides. Purified PCR product and pExu plasmid (Mancha-Agresti et al., 2016) were digested with the same restriction enzymes, as recommended by suppliers. The digested vector and insert were purified by Illustra^TM^ GFX^TM^ PCR DNA kit and then were ligated with T4 DNA ligase (Invitrogen, Carlsbad, CA, United States) and transformed into *E. coli* Top10, creating the *E. coli* Top10 (pExu:*mCherry*) strain. Sequence analysis was done to confirm the insert integrity using BigDye Terminator v3.1 Cycle Sequencing Kit (Applied Biosystems, Foster City, CA, United States) and ABI3130 sequencing equipment. Lastly, pExu:*mCherry* was transformed into competent cells of *L. lactis* ssp. *cremoris* MG1363 strain generating *L. lactis* MG1363 (pExu:*mCherry*) strain, used in this study.

### mCherry Production by Eukaryotic Cells *in vitro*

Chinese hamster ovarian cell line [Flp-In^TM^-CHO (Invitrogen)] (CRL 12023)-ATCC was used to evaluate the mCherry expression. For this intent, CHO cells were cultured in complete Nutrient Mixture F12 Ham media (Sigma-Aldrich), supplemented with 10% fetal bovine serum (Gibco-Thermo Scientific, Waltham, MA, United States), 1% L-glutamine (Sigma-Aldrich), and 1% HEPES (Sigma-Aldrich). Cells were seeded at 1 × 10^6^ in a 6-well plate and at 90–95% of confluence, CHO cells were transfected with 4 μg of pExu:*mCherry* vector or no plasmid (negative control) using Lipofectamine^TM^ 2000 (Invitrogen), according to supplier’s recommendation. Eukaryotic cells protein expression was checked by epifluorescent microscope (Zeiss Axiovert 200, filter 585/42 nm, Oberkochen, Germany) and by flow cytometry (BD FACSCanto^TM^, BD Bioscience, Franklin Lakes, NJ, United States). Duplicate transfection assays were performed.

### Bacterial Doses and Microencapsulation

To prepare the doses of *L. lactis* (pExu:*mCherry*), on the first day, the culture was grown at 30°C in M17 medium (Sigma-Aldrich) supplemented with 0.5% glucose (Labsynth) (GM17) and 125 μg/mL of erythromycin (Sigma-Aldrich). On the second day, a 1/20 dilution was performed until the culture reaches OD_600__nm_ = 1.0. Then 2 mL, which corresponds to one dose, centrifuged for 10 min at 4°C at 4,000 rpm. Supernatant was discarded, and the pellet was washed twice with PBS (0.01 M) and then it was resuspended in 30 μL of sterile PBS, immediately frozen in ultra-freezer −80°C until use. At the moment of gavage, sterile PBS (0.01 M) was added (∼70 μL) to the prepared doses to reach a final volume of 100 μL. The final quantities of administrated bacteria were 10^14^ CFU/dose. This concentration was calculated after adequate dilution (10^−14^), where colonies were counted by pour plate method (G-M17 agar-medium with erythromycin).

For encapsulated doses, after washing the pellet with PBS (0.01 M), bacteria culture [10^14^ colony forming unit (CFU)] was mixed with 50 μL of sterile PBS (0.01 M)and 50 μL of 1% of Sodium Alginate Solution matrix as described by Krasaekoopt et al. (2003). For this procedure, alginate solution was prepared and sterilized by autoclaving (121°C for 15 min). The mixture was homogenized carefully and this homogeneous solution (Bacteria+PBS+1% of sodium alginate solution) was extruded through a 21-gauge nozzle into a sterile 3% CaCl_2_ solution magnetically stirring (a cross-linking solution), forming beads (encapsulated bacteria) by contact of both solutions. Afterward, beads were harvested by filtering using Cell Strainer 40 μm Nylon (Corning, NY, United States) and CaCl_2_ remnants were removed by pipetting. Then, they were transferred to a sterile micro tube and stored in ultra-freezer −80°C until use. Before gavage, ∼100 μL of sterile PBS was added to frozen encapsulated doses.

### Encapsulation Efficiency and Viability After Freezing

To determine the encapsulation efficiency (EE), the beads were mixed with 100 μL of 2% (w/v) sterile sodium citrate buffer (pH = 8.36) during 10 min to dissociate the capsules. After this time, the mixture was vigorously homogenized and subsequently the entrapped viable bacteria, after adequate dilution (10^−10^), were counted by pour plate method (G-M17 agar-medium). Non-encapsulated bacteria were also submitted to the same treatment to guarantee the same experimental condition. This process was performed during 5 days after doses production to see viability of beads after freezing. Encapsulation efficiency after freezing was calculated by the following equation:

$$\text{EE}\%=\frac{\mathrm{CFU encapsulated cells}}{\mathrm{CFU non}-\mathrm{encapsulated cells}}\times 100$$

^∗^ CFU is a number of colony forming unit on the agar plate.

Efficiency was verified for freshly encapsulated and non-encapsulated bacteria (day 0) and for doses conditioned at −80°C (days 1 to 5). For the last condition, single doses were unfrozen and plated each day.

### Viability of Encapsulated Bacteria to Artificial Juice

To verify the viability of entrapped bacteria, stomach environment was simulated. In a 20 mL solution containing sodium chloride 0.9% (w/v) and 0.3% of pepsin (w/v) (Sigma-Aldrich) (pH = 2), 10^14^ CFU (one dose) of encapsulated and non-encapsulated bacteria was added and incubated for 2 h at 37°C, with shaking at 50 rpm (Chávarri et al., 2010). Afterward, both suspensions were centrifuged and the supernatant was discarded (the encapsulated solution was filtered using Cell Strainer 40 μm Nylon-corning to avoid capsule loss during discard of the supernatant). Then, the bacterial content was washed twice with PBS (0.01 M) and centrifuged in order to remove the remaining acid solution. Finally, pellets were ressuspended with 100 μL of sodium citrate buffer (pH = 8.36) and after that, 100 μL of this mixture was assayed to pour plate method. This experiment was performed in duplicate. The survival (%) of free and encapsulated bacteria was calculated by spread plate count on G-M17 agar after incubation at 30°C for 48 h as shown in the following equation:

(1) $$\begin{array}{l} \%\mathrm{Survival}=\\ \frac{\log\;(\mathrm{CFU beads after}\;2\;\mathrm{hours exposure to acid condition})}{\log\;(\mathrm{CFU initial beads count})}\times 100 \end{array}$$

### Mice

Conventional 4 to 6-week-old (25–30 g) male and female C57BL/6 mice, were obtained from Centro de Bioterismo (CEBIO) of Universidade Federal de Minas Gerais (UFMG, Brazil). Procedures and manipulation of animals followed the rules of Ethical Principles in Animal Experimentation, accepted by the Ethics Committee on Animal Experiments (Protocol # 114/2010, CETEA/UFMG/Brazil). All animals were maintained in collective cages in an environmentally controlled room with a 12-h light/dark cycle and given free access to water and food *ad libitum*.

### Mice Handling: Gavage of Recombinant *L. lactis* MG1363 Strain (Encapsulated and Non-encapsulated) Into C57BL/6 Mice

Mice were split into three groups as follows: (i) PBS group, (ii) non-encapsulated *L. lactis* ssp. *cremoris* MG1363 (pExu:*mCherry*), and (iii) encapsulated *L. lactis* MG1363 (pExu:*mCherry*). Two independent experiments were performed with 24 mice in each group (72 mice/experiment). The animals were separated according to each evaluated time (three animals for each time). The recombinant strains (10^14^ CFU bacterial suspensions in a final volume of 100 μL of PBS) were orally administered by gavage. The administration was performed in one go (at zero time) and, at the following intervals: 12, 24, 48, 72, 96, 120, 144, and 168 h (post-gavage), animals were euthanized by cervical dislocation and intestinal sections of duodenum, jejunum, ileum and colon were analyzed by confocal microscopy and qRT-PCR. Images were captured using Zeiss LSM 540 META inverted confocal laser-scanning microscope and analyzed by Zeiss LSM Image Browser software.

### mRNA Extraction and Real-Time PCR (qRT-PCR)

Samples of intestinal sections (duodenum, jejunum, ileum, and colon) at 24 h post-gavage, were collected to perform qRT-PCR assay to confirm mCherry expression in enterocytes after oral gavage. We even collected intestinal samples (duodenum, jejunum, ileum, and colon) at different times post-gavage (12, 48, 72, 96, 120, 144, and 168 h). Samples from each gut portion were collected after mice euthanasia and stored at −20°C in RNA later (Invitrogen) until RNA extraction. Total RNA was obtained using TRIzol reagent (Invitrogen), in compliance with manufacturer instructions. The quality and quantity of RNA samples were evaluated in agarose gel electrophoresis, and also through spectrophotometer analysis on NanoDrop^®^ 2000 spectrophotometer (Thermo Scientific) taking into account absorbance ratios of 280/260 and 260/230 nm. Extracted RNA samples were treated with DNAse I (Invitrogen) which was subsequently deactivated. The complementary deoxyribonucleic acid (cDNA) synthesis was performed using 1 μg of RNA and ImProm-II reverse transcriptase (Promega, Madison, WI, United States) in compliance with its manual.

Quantitative reverse transcription PCR (qRT-PCR) was performed using iTaq Universal SYBR Green Supermix (Bio-Rad, Hercules, CA, United States) and gene specific-primers for *mCherry* open reading frame (ORF). Housekeeping gene *Actb* was used as reference for normalization (Giulietti et al., 2001). Experimental approach was optimized by adjusting concentrations of primers for optimal specificity and efficiency (**Table 2**). Amplification reactions were performed in final volume of 10 μL, using 5 μL of SYBR green supermix and 10 ng of cDNA (5 ng/μL). The PCR cycle parameters were as follows: initial denaturation at 95°C for 30 s, annealing/extension at 60°C for 60 s, 40 cycles of 95°C for 15 s followed by a dissociation stage for recording the melting curve. Expression levels in control group were used as calibration data (i.e., animals which received PBS). Results were shown graphically as fold changes in gene expression, using the means and standard deviations of target gene expression amount in accordance with Hellemans et al. (2007). Data were analyzed according to the relative expression using 2^−ΔΔCt^ method (Hellemans et al., 2007).

**Table 2:** Targets and primers concentration of qRT-PCR performed in this study.

RNA target	Primer sequence (5′–3′)			
	Forward	Reverse	Amplicon size (pb)	Primer (μmol l^−1^)
mCherry	CACTACGACGCTGAGGTCAA	GTGGGAGGTGATGTCCAACT	97	0.25
β-Actin	AGAGGGAAATCGTGCGTGAC	CAATAGTGATGACCTGGCCGT	138	0.25

### Statistical Analysis

Statistical analysis was performed using GraphPad Prism 6. Statistical significance between the groups was calculated using a Student’s *t*-test. A 95% confidence limit was significant at a value of *P* < 0.05.

## Results

### Recombinant Strain of *L. lactis* – *L. lactis* MG1363 (pExu:*mCherry*)

The mCherry ORF (711bp) (GenBank No. AST15061.1) was successfully cloned into the pExu vector (Mancha-Agresti et al., 2016). This vector has an eukaryotic unit harboring the cytomegalovirus mammalian promoter (pCMV), the polyadenylation signal of the bovine growth hormone (BGH), and a prokaryotic region containing RepD/RepE replication origins for *L. lactis* and also OriColE1 replication origin for *E. coli*. The resistance marker Erythromycin (Ery), was used to select recombinant strain. pExu:*mCherry* vector construction was successfully confirmed by molecular techniques: PCR, enzymatic digestion (*Not*I and *Xho*I), and also DNA sequencing. pExu:*mCherry* was transformed into *L. lactis* ssp. *cremoris* MG1363 wild type strain generating *L. lactis* MG1363 (pExu:*mCherry*) recombinant strain.

### Eukaryotic Cells Are Able to Express the mCherry Protein

**FIGURE 1:**
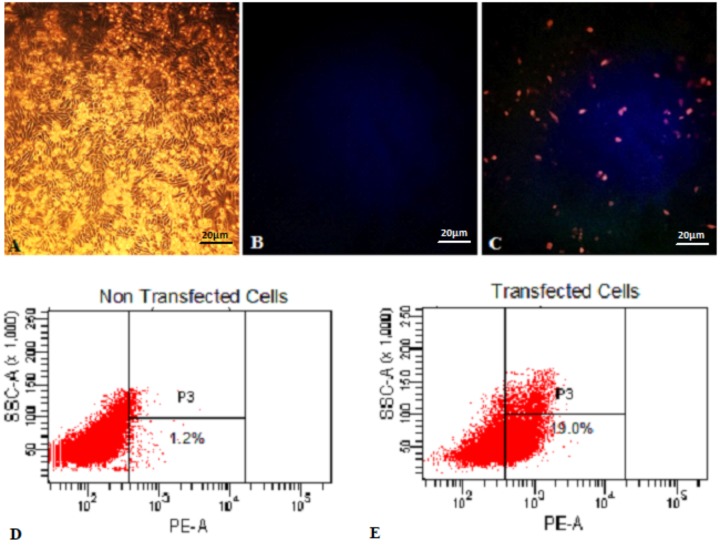
Expression of mCherry protein by Eukaryotic cells transfected with pExu:*mCherry* plasmid evaluated by Fluorescence Microscopy and Flow Cytometry. **(A)** Light field capture of non-transfected CHO cells, **(B)** non-transfected CHO cells, **(C)** transfected CHO cells with pExu:*mCherry* (20X), **(D)** Dot plot of non-transfected cells, **(E)** Dot plot transfected cells with pExu:*mCherry*. The fluorescence images were obtained using the epifluorescent microscope (Zeiss Axiovert 200, filter 585/42 nm). The dot plot graph shows the cell count on the y axis and the PE-A filter on the x axis examined through BD FACSDiva^TM^ Software-BD-biosciences.

Functionality of pExu:*mCherry* was established after transfection of CHO cells in two independent assays. In the first one, fluorescence microscopy analysis showed that transfected eukaryotic cells were able to express the reporter protein. In contrast, non- transfected cells showed no fluorescence, as expected. In **Figure 1**, the pictures A, B, and C show the eukaryotic cell expressing or not the fluorescent protein. Images were captured by fluorescence microscopy (**Figures 1A–C**).

In the second one, the percentage of expressing cells was evaluated by flow cytometry. After 48 h, 19% of transfected cells with pExu:*mCherry* showed ability to express the reporter protein. No expression was observed in the non-transfected cells, and also in cells transfected with pExu:*empty* (data not shown). In **Figure 1** pictures D and E show the dot plot of non-transfected and transfected cells respectively. These results confirmed functionality of pExu:*mCherry* plasmid in eukaryotic cells.

### Encapsulation Efficiency in 1% (w/v) Alginate and Loss of Viability After Encapsulation and Freezing of *L. lactis* MG1363 (pExu:*mCherry*)

At first, alginate concentration for encapsulation was standardized by testing three different concentrations (0.5, 1, and 2% w/v) with 3% CaCl_2_ (w/v). Results obtained at this stage showed that the uniformity and spherical beads at 0.5% (w/v) of alginate concentration were not satisfactory due to low viscosity and consequently, less number of binding sites for Ca^2+^ ions (cross-linkage) (Chandramouli et al., 2004; Lotfipour et al., 2012). With 2% (w/v) alginate concentration, beads were too viscous to be extruded from the 21G needle. Thus, the 1% (w/v) alginate concentration presented the best conditions for encapsulation.

**FIGURE 2:**
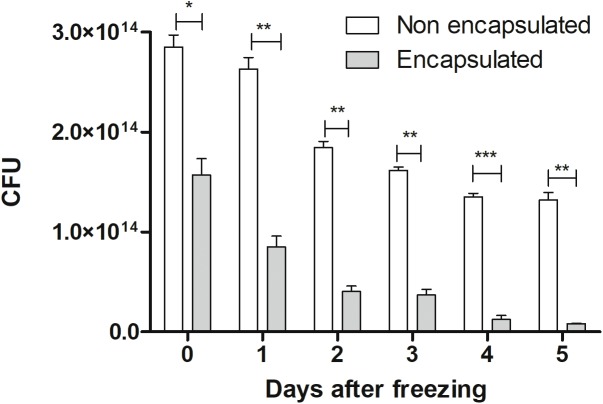
Cellular viability: viability of non-encapsulated and encapsulated cells of *L. lactis* MG1363 (pExu:*mCherry*) from 0 to 5 days after freezing (–80°C). The 0 represents the number of CFU by plate count immediately after the encapsulation process. ^∗^*P* < 0.05, ^∗∗^*P* < 0.001, and ^∗∗∗^*P* < 0.0001.

Regarding efficiency of encapsulation, our results showed that at the time of doses preparation it had 2.8 × 10^14^ CFU. After the encapsulation process (represented as 0 time), we observed a decrease in the number of CFU (1.6 × 10^14^), being about 60% efficiency, in comparison with non-encapsulated ones. It was possible to observe that both strains (encapsulated and non-encapsulated) lost viability during freezing days, in equivalent form, as demonstrated in **Figure 2**. Therefore, in this study, only recent confectioned doses were used in oral administration to mice.

In artificial gastric juice, our results showed that the number of cultivable cells observed corresponds to 4.5 × 10^4^ CFU and 4.7 × 10^4^ CFU for non-encapsulated and encapsulated, respectively. Regarding initial doses in non-encapsulated bacteria (2.8 × 10^14^ CFU) and encapsulated (1.6 × 10^14^ CFU), we can observe that the percentage survival of non-encapsulated bacteria was 25.3%, while the survival of encapsulated strain was 32.9%. The bacterial survival showed around an 8% increase in encapsulated ones. These results lead to the conclusion that encapsulated bacteria has slightly more resistance to survive in artificial gastric juice, demonstrating that encapsulate process can be successfully used in oral administration strategies.

### mRNA and Protein Expression in Mice Bowel

**FIGURE 3:**
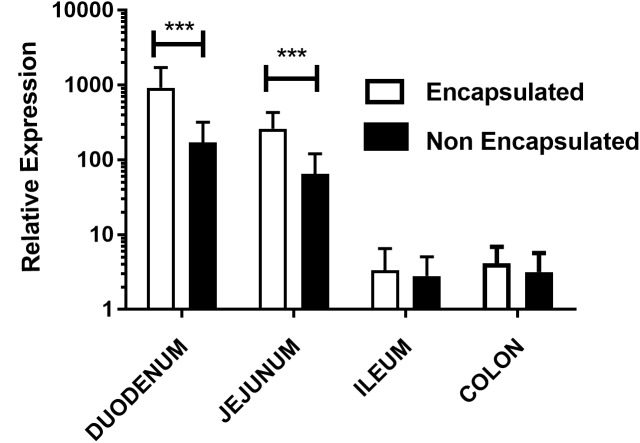
Relative expression of mCherry protein in mouse intestinal cells: evaluation of mCherry protein expression by epithelial cells from different sections of the gut of mice treated orally with, non-encapsulated or encapsulated, *L. lactis* MG1363 (pExu:*mCherry*), by the qRT-PCR technique. Axis y shows the relative expression, and axis x shows the gut sections. ^∗∗∗^*P* < 0.0001.

In order to investigate the expression of mCherry protein by eukaryotic cells *in vivo*, we evaluated mRNA expression of mCherry in different parts of the bowel, after gavage. Our results showed that animals which received encapsulated bacteria were able to express 4.34x fold increase in duodenum and 3.03x fold in jejunum portion when compared with non-encapsulated bacteria. However, no significant differences were seen in ileum and colon portions as shown in **Figure 3**. When the kinetics qRT-PCR experiments were performed, it was observed the same behavior as in confocal analysis. Animals which received the encapsulated doses have higher relative expression in duodenum and in jejunum sections while in ileum and colon the relative expression was lower. Nevertheless, in the colon no statistical differences were observed. The same behavior was observed in the relative expression of non-encapsulated bacteria, where the levels of expression in the duodenum and in the jejunum sections were higher compared to ileum and colon ones (**Supplementary Figures 1**, **2**).

### Intestinal Cells of Mice Orally Administered With Encapsulated *L. lactis* MG1363 (pExu:*mCherry*) Showed mCherry Expression in Distant Regions of the Bowel

**FIGURE 4:**
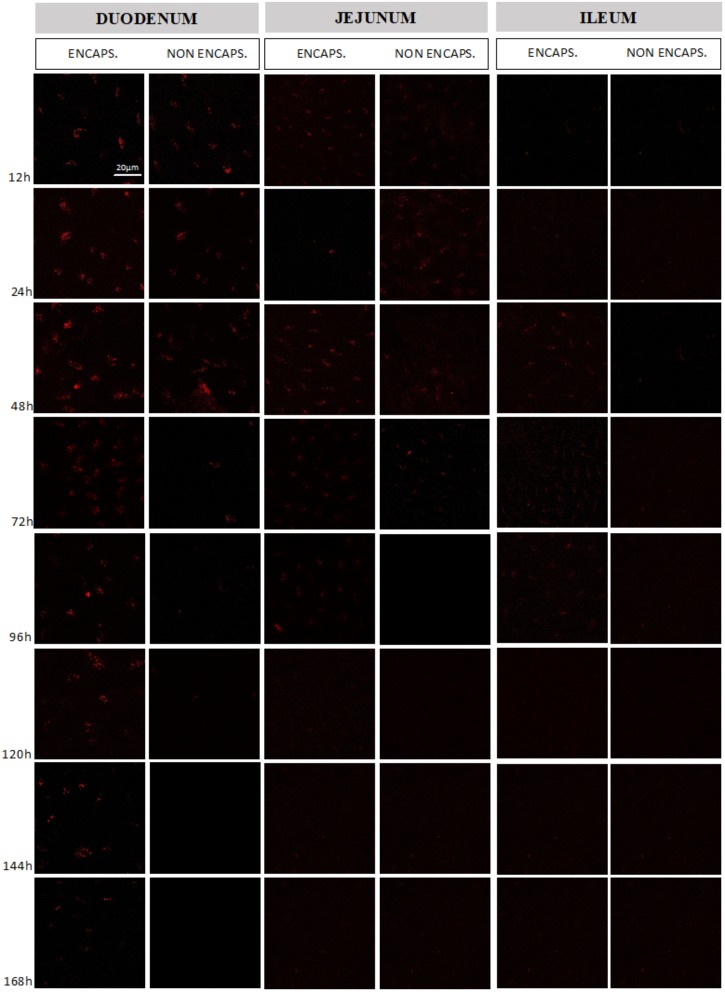
Evaluation of mCherry protein expression by mouse intestinal cells. Comparison between the expression obtained in the different intestinal sections (duodenum, jejunum, and ileum) from the delivery of the vector pExu:*mCherry* by non-encapsulated and encapsulated bacteria. The expression was evaluated between 12 and 168 h after mice gavage. The images were obtained using Zeiss LSM 510 META inverted confocal laser-scanning microscope and the images were analyzed by Zeiss LSM Image Browser software.

After oral administration of encapsulated and non-encapsulated *L. lactis* MG1363 (pExu:*mCherry*) to C57BL/6 mice at different times (12, 24, 48, 72, 96, 120, 144, and 168 h), the expression of mCherry protein by eukaryotic cells was investigated. The eukaryotic cells of the duodenum and jejunum were able to express mCherry protein until 3 days post-gavage with non-encapsulated bacteria as was shown in previous report with eGFP protein (Mancha-Agresti et al., 2016), while encapsulated bacteria was able to keep the protein expression for 7 and 4 days in duodenum and jejunum, respectively. When we investigated the ileum and colon sections, there was no expression found after gavage with non-encapsulated bacteria. Nevertheless, ileum of animals which received the encapsulated *L. lactis* expressed the recombinant protein between 2 and 4 days after gavage, as shown in **Figure 4**. In the colon section no expression was found (data not shown). The confocal microscopy showed two important aspects. First, there was more expression of mCherry protein by eukaryotic cells when encapsulated *L. lactis* was administered, as were shown in qRT-PCR results. The other aspect is the encapsulated strain is able to reach distant parts of the bowel, as ileum, for example (**Figure 4**).

## Discussion

*Lactococcus lactis* is commonly studied as a host for heterologous protein production and as a delivery platform for therapeutic molecules (Morello et al., 2008; Bahey-El-Din et al., 2010). Different strategies have been proposed in order to improve the delivery in the gut, including the membrane fragilization (Tao et al., 2011) or the expression of invasin proteins in the bacteria membrane (Innocentin et al., 2009). Even though these strategies have drawbacks, many reports showed delivery efficiency when using the DNA vaccine platform, by both oral (Zurita-Turk et al., 2014; Pereira et al., 2015; Souza et al., 2016) or nasal administration (Mancha-Agresti et al., 2017).

The eGFP expression by enterocytes, after mice oral administration of non- encapsulated *L. lactis* MG1363 (pExu:*egfp*), was successfully reported by our group (Mancha-Agresti et al., 2016). It was also reported that delivery by this recombinant strain only occurred in the duodenum section between 12 and 72 h after gavage. The eGFP protein is the marker of choice for many biological studies. However, there are limitations to its use, both *in vivo* and *in vitro* experiments. For example, GFP may lose its direct fluorescence during tissue fixation, as well as for subsequent processing which leads to commercial use of antibodies for immunostaining for GFP detection. Also, GFP expression shows substantial biological variability, even among similar cell types in a single animal (Anderson et al., 2005; Brazelton and Blau, 2005). In addition, GFP protein could have diminished brightness, variability, or loss of fluorescence due to auto-fluorescence of the cells in green wavelengths, and also a delay between protein synthesis and fluorescence development (Cubitt et al., 1995; Aspiras et al., 2000; Shaner et al., 2005). Even more, conventional fluorescent proteins, such as GFP, emit in visible spectrum, where light absorption by tissue is strong (Deliolanis et al., 2008), therefore, its sensibility is low.

In order to improve our previous results and circumvent the drawbacks of *egpf* or *gfp* reporter gene, we decided to evaluate another reporter, the *mCherry*, using the same system, the pExu vector delivered by *L. lactis* ssp. *cremoris* MG1363. The mCherry is a monomeric, non-toxic protein and doesn’t need any stress induction to host organisms expressed at high levels (Carroll et al., 2010). It is highly resistant to photobleaching, consequently losing less fluorescence overtime (Viegas et al., 2007). An important issue to highlight about mCherry in comparison with other red-shifted fluorescent proteins, is their fluorescence emission beyond the 600 nm spectra, where light absorption by tissues is substantially reduced, this showcases enhanced detection sensitivity in whole body imaging applications by at least two orders of magnitude over GFP (Deliolanis et al., 2008).

A previous study using mCherry reporter gene was conducted by van Zyl et al. (2015) to analyze, after mice gavage, the migration and colonization in the gastrointestinal tract (GIT) of two recombinant LAB: *Enterococcus mundtii ST4SA* and *Lactobacillus plantarum 423.* This study reports mCherry as a promising reporter system for living cell imaging studies *in vitro*, *in vivo*, and *ex vivo.* They showed that both bacteria predominantly colonized the cecum and colon, and also persisted in the gut for at least 24 h (van Zyl et al., 2015). Another study used *mCherry* ORF cloned in prCR12 vector (heterologous expression of mCherry protein), transfected in three strains of *Lactobacillus* (*L. plantarum* Lp90, *L. plantarum* B2, and *L. fermentum* Pbcc11.5) showed great strategy in estimating gut colonization in zebrafish model using probiotics. Another interesting point highlighted by these authors is to avoidance of animal sacrifice (Russo et al., 2015).

Acid gastric secretion, a major defense mechanism against the majority of ingested microorganisms, is the first stress confronted by orally administered bacteria (Marteau et al., 1997). Once *L. lactis* has overcome the gastric barrier, they need to envisage the enteric secretions encountered in the duodenum region such as mucus, lysozyme, and phospholipase A2 (Peeters and Vantrappen, 1975; Harwig et al., 1995), defensins secreted by Paneth cells (Mallow et al., 1996) and also bile salt and pancreatic juices. Due to these adversities, high cell mortality is detected in duodenum (Drouault et al., 1999). Divergence may occur once distinct bacterial species demonstrate different reductions in viability, as shown in a study comparing survival of *Bifidobacterium bifidum*, *Lactobacillus acidophilus*, *Lactobacillus bulgaricus*, and *Streptococcus thermophilus* in the gastric compartment (Marteau et al., 1997).

To prevent this situation, and consequently increase *L. lactis* viability in duodenum region and also reach further regions from the intestine, we investigated the encapsulation technique, that has been extensively studied and standardized in *in vitro* experiments (Sheu and Marshall, 1993; Rowley et al., 1999; Krasaekoopt et al., 2003; Desai and Jin Park, 2005; Urbanska et al., 2007; Nedovic et al., 2011).

Once the focus of our group is the use of LAB as DNA vaccine platform in the treatment of different gut disease, like inflammatory bowel disease (IBD), an important issue concern how the encapsulation doses could be affected by the freezing. Demonstrating that bacteria remains viable after freezing could help the development of new therapeutical strategies, for example, preparation of encapsulate doses in one moment and administrate them in another one. Even more, in developing countries this application could help to solve logistic issues, for example frozen doses could be transport further distances without big damage. In order to provide support for *in vivo* studies, we initially performed *in vitro* assays to verify the efficiency of encapsulation process with alginate matrix and the viability of bacteria doses for 5 days after freezing.

In contrast with Kailasapathy (2006) studies, in which EE of *Lactobacillus acidophilus* and *Bifidobacterium lactis* was tested after storage, we unexpectedly verified a decrease in the CFU of *L. lactis* ssp. *cremoris* MG1363 doses soon after the encapsulation process when compared with non-encapsulated. However, when these bacteria were submitted to low pH conditions, simulating gastric environment, the viability of encapsulated bacteria increased around 8% when compared with non-encapsulated. The important issue of this result is to highlight that encapsulation matrix was able to protect the bacteria against these astringent conditions.

Our findings are in accordance with Mortazavian et al. (2007) who tested three different pH (extreme, intermediate, and normal), in extreme and normal conditions they showed that encapsulated *L. acidophilus*, with 2% sodium alginate, had slightly higher percentages of survival than non-encapsulated, being 17.4 and 16%, respectively (Mortazavian et al., 2007). Also, an increase of 9.52 and 7.52% was described in the survival of encapsulated bacteria (*L. acidophilus)* with again 2% of sodium alginate at pH = 3.0 for 3 h in 2 and 1% of bile salt respectively (Chandorkar et al., 2018). These authors highlighted that microencapsulation is a competent technique able to protect probiotics against gastrointestinal environment. All this data supports the idea that encapsulated techniques improve the survival of probiotic bacteria against unfavorable conditions.

This finding was corroborated by our *in vivo* results where the expression of mCherry protein in the duodenum was higher for encapsulated bacteria and was also detected in ileum. The qRT-PCR provided quantitative results that were in line with those obtained in confocal microscopy, specially, in duodenum and jejunum portions, evidencing higher expression of mCherry protein in animals which received encapsulated doses. Although fluorescence had been observed by confocal microscopy at ileum portion, no significant differences were observed in qRT-PCR assay for both, ileum and colon.

Microencapsulation process shows to protect bacteria doses against environment through the GIT. Our hypothesis is that the alginate matrix protects the bacteria against the adversities (stomach and duodenum), allowing their movement across the bowel, being the bacteria able to deliver the plasmid in further sections, as jejunum, ileum, and also slightly in colon.

To our knowledge, this is the first study describing the comparison of encapsulated vs. non-encapsulated *L. lactis* using DNA delivery strategy, *in vivo*. Compared with our previous results (Mancha-Agresti et al., 2016) microencapsulation technique enables higher and longer recombinant protein expression in the gut. Furthermore, this process increases bacteria survival allowing further segments of the gut such as ileum to be reached. The methodology of encapsulation adopted in this work is able to enhance the potential for mucosal delivery by LAB. These findings are uplifting for mucosal therapy for different kinds of bowel diseases.

## Author Contributions

NC-R, MD, CC, LJ, and PM-A conceived the project. NC-R, MD, PM-A, and SCS wrote the original draft of the manuscript. NC-R, MD, PM-A, SL, AN, and VA wrote, reviewed and edited the manuscript. VA obtained the funding and supervised the project.

## Conflict of Interest Statement

The authors declare that the research was conducted in the absence of any commercial or financial relationships that could be construed as a potential conflict of interest.
