# Supplementary material for: Microencapsulation of Lactic Acid Bacteria Improves the Gastrointestinal Delivery and in situ Expression of Recombinant Fluorescent Protein
Source: Front Microbiol. 2018 Oct 5;9:2398. doi: 10.3389/fmicb.2018.02398 (PMC6182071; doi:10.3389/fmicb.2018.02398)
Supplement: Supplementary file 1 [file Data_Sheet_1.docx]

**Supplementary Material**

**Microencapsulation of Lactic Acid Bacteria improves the gastrointestinal delivery and in situ expression of recombinant fluorescent protein**

Nina Dias Coelho-Rocha^1^, Camila Prosperi de Castro^1,2^, Luis Cláudio Lima de Jesus^1^, Sophie Yvette Leclercq^3^, Savio Henrique de Cicco Sandes ^4^, Alvaro Cantini Nunes^4^, Vasco Azevedo^1^, Mariana Martins Drumond^1,5†^ and Pamela Mancha-Agresti^1†^.

1. Laboratório de Genética Celular e Molecular (LGCM), Instituto de Ciências Biológicas, Departamento de Biologia Geral, Universidade Federal de Minas Gerais (UFMG), Belo Horizonte, Minas Gerais, Brazil.
2. Kroton Educacional, Faculdade Pitágoras, Contagem, Minas Gerais, Brazil.
3. Laboratório de Inovação Biotecnológica, Fundação Ezequiel Dias (FUNED), Belo Horizonte, Minas Gerais, Brazil.
4. Laboratório de Genética Molecular de Protozoários Parasitas, Instituto de Ciências Biológicas, Departamento de Biologia Geral, Universidade Federal de Minas Gerais (UFMG), Belo Horizonte, Minas Gerais, Brazil.
5. Centro Federal de Educação Tecnológica de Minas Gerais (CEFET/MG), Coordenação de Ciências, Belo Horizonte, Minas Gerais, Brazil.

†These authors have contributed equally to senior authorship.

# *Correspondence author

mmdrumond@gmail.com

vasco@icb.ufmg.br

p.mancha.agresti@gmail.com

**The Kinetics qRT-PCR**

**Non-Encapsulated bacteria Encapsulated bacteria**


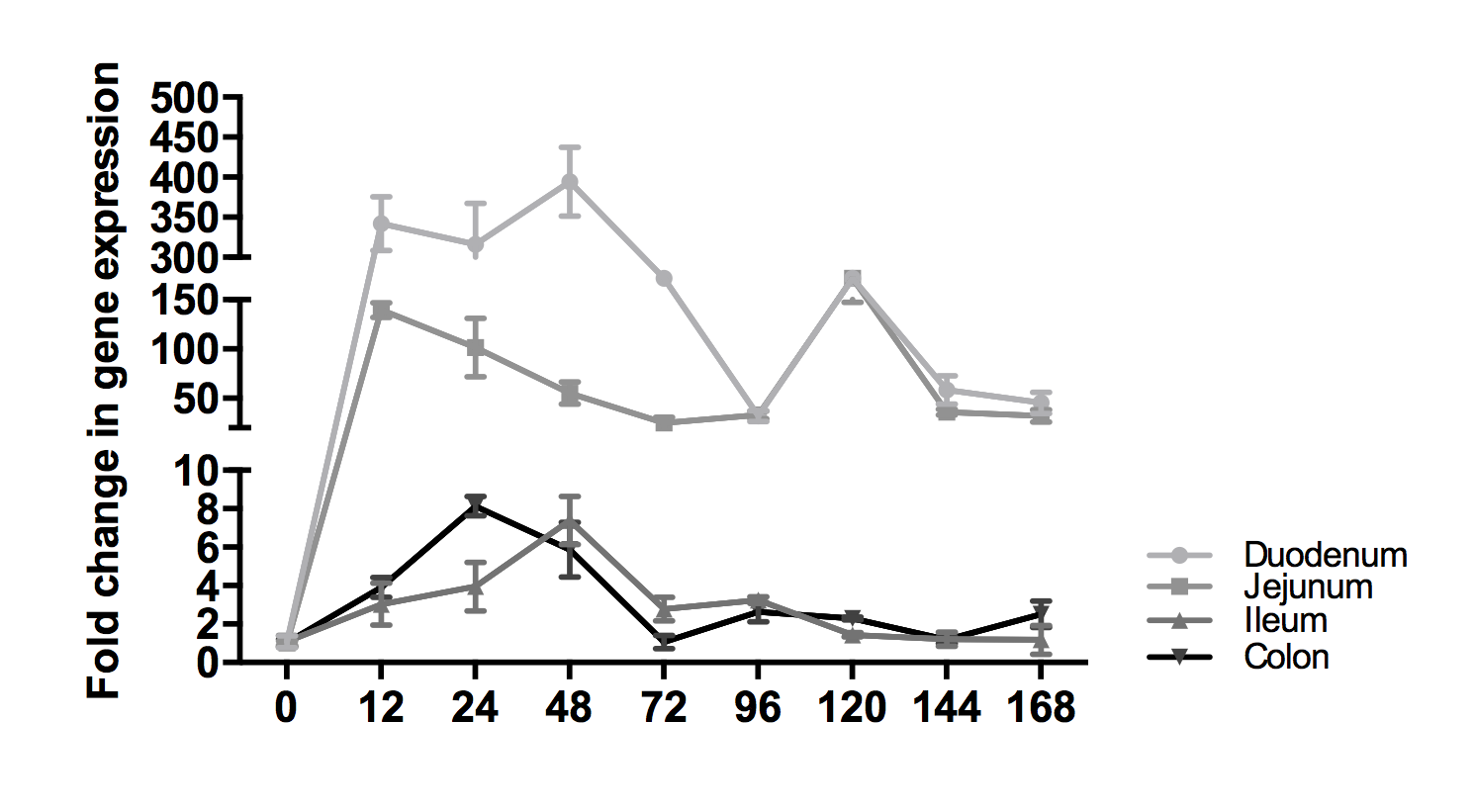

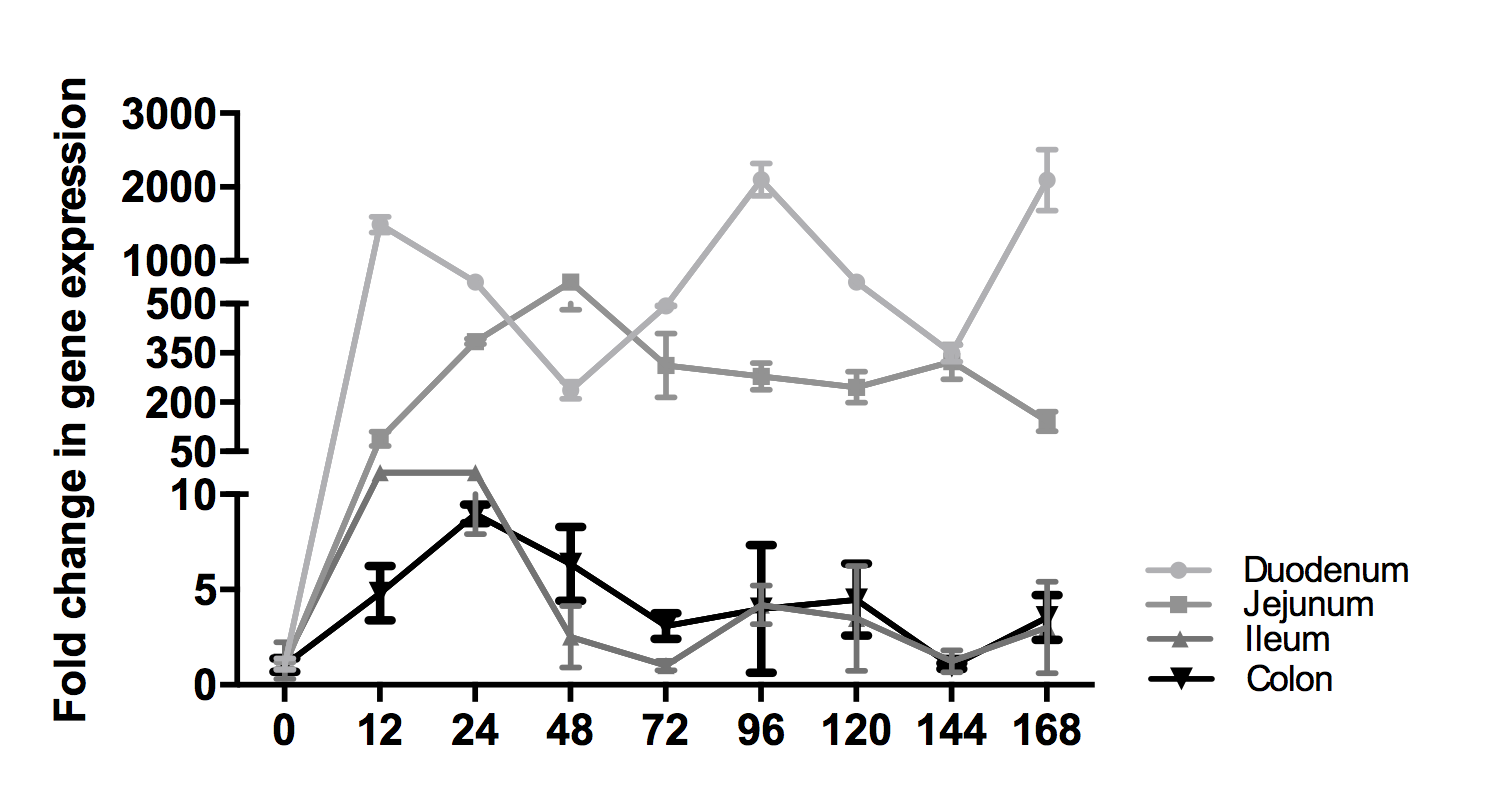


**Supplementary Figure 1**: **Kinetic of** **mCherry expression by Quantitative Real-time in mouse eukaryotic cells:** Evaluation of mCherry protein expression by epithelial cells from different sections of the gut (duodenum, jejunum, ileum and colon) at different times (0, 12, 24, 48, 72, 96, 120, 144 and 168 hours) post-gavage of mice treated orally with *L. lactis* MG1363 (pExu:*mCherry*) non-encapsulated (A) or *L. lactis* MG1363 (pExu:*mCherry*) encapsulated (B), by the qRT-PCR technique. Axis Y shows the relative expression, and axis X shows hours post-gavage.

**Supplementary Figure 2**: **Relative expression comparison of mCherry protein from different sections of the mice bowel:** Comparison of mCherry protein expression by epithelial cells from different sections of the gut (duodenum, jejunum, ileum and colon) at different times (0, 12, 24, 48, 72, 96, 120, 144 and 168 hours) post-gavage of mice treated orally with, non-encapsulated and encapsulated, *L. lactis* MG1363 (pExu:*mCherry*), by the qRT-PCR technique. Axis Y shows the relative expression, and axis X shows hours post-gavage *P<0.01 ,** P<0.001.
